# Nicotinamide phosphorybosiltransferase overexpression in thyroid malignancies and its correlation with tumor stage and with survivin/survivin DEx3 expression

**DOI:** 10.1007/s13277-015-3506-z

**Published:** 2015-05-07

**Authors:** Nadia Sawicka-Gutaj, Joanna Waligórska-Stachura, Mirosław Andrusiewicz, Maciej Biczysko, Jerzy Sowiński, Jerzy Skrobisz, Marek Ruchała

**Affiliations:** 1 Department of Endocrinology, Metabolism and Internal Medicine, Poznan University of Medical Sciences, Przybyszewski St. 49, Poznań, Poland; 2 Department of Cell Biology, Poznan University of Medical Sciences, Rokietnicka St. 5d, Poznań, Poland; 3 Department of General, Gastroenterological and Endocrine Surgery, Poznan University of Medical Sciences, Przybyszewski St. 49, Poznań, Poland; 4 Department of General Surgery and Multiple Trauma, with Division of Gastroenterological and Endocrine Surgery, Provincial Hospital, Juraszów St. 7/19, Poznań, Poland

**Keywords:** Thyroid gland, Thyroid cancer, Nodular goiter, Nicotinamide phosphoribosyltransferase, Visfatin, Reverse transcriptase polymerase chain reaction

## Abstract

Nicotinamide phosphorybosiltransferase (NAMPT) plays an important role in the regulation of cellular growth, angiogenesis, and apoptosis in mammalian cells. *NAMPT* overexpression has been recently found in colorectal, breast, prostatic, gastric, esophageal, pancreatic cancers, and specific NAMPT inhibitors might be adjuvant therapeutic modalities. In this study, we analyzed *NAMPT* expression in 40 malignant and in 67 benign thyroid tissue samples using qPCR. We also investigated relationships between *NAMPT* expression and survivin/survivin splicing variants DEx3 and 2B expressions. *NAMPT* expression was significantly higher in thyroid cancers (*P* < 0.0001), and it was positively correlated with tumor stage (*P* = 0.0012; *r* = 0.493). *NAMPT* expression was significantly higher in tumors staged pT3 or pT4 (16 cases) than in tumors staged pT1 or pT2 (24 cases) (*P* = 0.0106). Metastases to the lymph nodes were found in 12 out of 40 cases, and *NAMPT* expression was higher in the metastatic group (*P* = 0.0258). Multifocality was not associated with higher *NAMPT* expression (*P* = 0.3451). *NAMPT* expression in thyroid cancers significantly correlated with survivin and with survivin splice variant DEx3 expressions (*P* < 0.0001; *r* = 0.624 and *P* = 0.0239; *r* = 0.357, respectively). There was no correlation between *NAMPT* and survivin 2B expressions (*P* = 0.3508). This is the first study demonstrating *NAMPT* overexpression in thyroid malignancies using quantitative RT-PCR. Moreover, it shows that NAMPT is upregulated in patients with more advanced tumor stage and metastatic disease which may prove to be clinically relevant. Further studies are needed to explain the role of NAMPT in thyroid cancer biology and the possible use of NAMPT inhibitors in thyroid cancer.

## Introduction

Nicotinamide phosphorybosiltransferase (NAMPT), also known as visfatin or pre-B-cell-enhancing factor is a rate-limiting enzyme catalyzing the synthesis of cellular nicotinamide adenine dinucleotide (NAD+), which is required for ATP production. NAMPT is involved in energy homeostatis in tumor cells. Also, NAMPT activity has been shown to play an important role in de novo lipogenesis in prostate cancer cells [1]. Altogether, NAMPT plays an important role in the regulation of cellular growth, angiogenesis, and apoptosis in mammalian cells. *NAMPT* overexpression has been recently found in colorectal, breast, prostatic, gastric, esophageal, and pancreatic cancers [2–4]. The downregulation of NAMPT promotes apoptosis in cancer cells and attenuates tumor growth [5, 3]. Moreover, inhibition of NAMPT used in combination therapy sensitizes tumor cells to genotoxic agents or radiotherapy [6]. Insight into the mechanism behind NAMPT role in tumorigenesis led to the development of specific NAMPT inhibitors that might have a future role in adjuvant therapeutic modalities in cancers not responding for the conventional therapy [5, 7]. Despite the initial adverse effects associated with general cytotoxicity of NAMPT inhibitors, the highly specific compound APO866 acting as a noncompetitive inhibitor of NAMPT was developed in 2003 [8]. Furthermore, three NAMPT inhibitors APO866, GMX1777, and GMX1778 have been evaluated in clinical trials but their efficacy is not sufficient due to on-target toxicities [9]. APO866 also inhibits NAMPT activity in inflammatory cells such as macrophages, neutrophils, and monocytes [10–13]. Therefore, it has been recognized as an anti-inflammatory agent effective in arthritis, myocardial infarction, atherosclerosis, and cryoinjury of the brain [14, 15, 10, 16].

Recently, thyroid cancer incidence has increased significantly in developed countries [17]. Thyroid cancers arising from the follicular epithelium are categorized as either differentiated thyroid cancers (DTC) (papillary, follicular, Hürthle cell carcinomas) or anaplastic cancer. Medullary thyroid cancer is derived from parafollicular C cells. Standard treatment for DTC includes thyroidectomy, radioiodine ablation, and TSH suppression with levothyroxine [18, 19]. Most patients respond well to therapy; however, about 10–15 % of patients with disseminated differentiated thyroid cancer develop radioiodine refractory disease, and novel therapeutic modalities are warranted in this group [20]. Similarly, patients with advanced medullary thyroid carcinoma treated with tyrosine kinase inhibitors experience only partial benefit and they have a low chance for complete response [21]. Therefore, studies on molecular regulation of thyroid cancers are essential.

Herein, we analyzed *NAMPT* expression in malignant and benign thyroid lesions in the context of clinicopathological data. In addition, we correlated *NAMPT* expression with the expression of another anti-apoptotic protein—survivin and its splice variants 2B and DEx3. Survivin overexpression is characteristic for many malignancies including thyroid cancer, and it appears to be associated with tumor progression as we demonstrated in our previous study [22, 23]. Survivin and survivin DEx3 have anti-apoptotic and cytoprotective properties, while survivin 2B attenuates their action and is considered as a pro-apoptotic protein [24]. Survivin DEx3 overexpression was reported in many malignancies, and it correlates with tumor aggressiveness and poor prognosis [22].

## Materials and methods

From 2012 to 2014, 107 samples of newly diagnosed and surgically treated nodular goiters or thyroid cancers were collected. There were 40 tissue samples of thyroid cancers: 29 of papillary thyroid cancer, 5 of medullary thyroid cancer, 4 of undifferentiated thyroid cancer, and 2 of follicular thyroid cancer. Thirty-five cases of benign lesions including 26 cases of colloid nodules, 5 cases of follicular adenomas, 4 cases of hyperplastic thyroid nodules, and 32 cases of healthy thyroid tissue derived from healthy regions of thyroid removed due to the cancer or the nodular goiter served as the control group. Resected thyroid tissues were immediately submerged in RNA protective medium and stored at −80 °C until qPCR analysis. The study was approved by the ethics committee of Poznan University of Medical Sciences, and informed written consent was obtained from each patient.

### cDNA synthesis and qPCR

A cDNA library was constructed as described previously [25]. To assess the expression level of *NAMPT* [NCBI: NM_005746.2] and survivin gene’s splice variants, *BIRC5* [NCBI: NM_001168], *BIRC5*-DEx3 [NCBI: NM_001012270.1], *BIRC5*-*2B* [NCBI: NM_001012271.1], and *HPRT* reference gene [Human HPRT Gene Assay Cat. No. 05 046 157 001 (Roche Diagnostics, Manheim, Germany)], qPCR with sequence specific primers and TaqMan hydrolysis probes from the collection of Universal Probe Library (Roche) was applied (Table 1). TaqMan probes were designed using ProbeFinder Software (version 2.50).

**Table 1:** Primers and the TaqMan hydrolysis probes used in this study

Gene	TaqMan probe no.	Forward primer 5′–3′	Reverse primer 5′–3′	Amplicon
*NAMPT*	#6 (Cat. No. 04685032001	aagggatggaactacattcttgag	ctgtgttttccaccgtgaag	118 bp
*total BIRC5*	#36 (Cat. No. 04687949001)	gcccagtgtttcttctgctt	aaccggacgaatgcttttta	88 bp
*BIRC5*-*ΔEx3*	#36 (Cat. No. 04687949001)	cagtgtttcttctgcttcaagg	cttattgttggtttcctttgcat	77 bp
*BIRC5*-*2B*	#36 (Cat. No. 04687949001)	tctgcttcaaggagctgga	aaagtgctggtattacaggcgta	88 bp
*HPRT*	Human *HPRT* Gene Assay (Cat. No. 05 046 157 001 (Roche))			

qPCR reactions were conducted in a reaction volume of 20 μl according the LightCycler^®^ TaqMan^®^ Master manufacturer’s protocol (Roche). Standard curves, to calculate the PCR reactions efficiencies, were constructed with decimal dilution of the cDNA library from OVCAR3 cell line (ATCC^®^). The PCR results were assembled using the LCDA Software version 4.0.5.415 dedicated for the LightCycler^®^ 2.0 instrument (Roche).

Relative expression of analyzed genes was normalized against *HPRT* gene. Each reaction was conducted in duplicate using newly synthesized cDNA and involved negative, nontemplate control.

### Statistical analysis

Statistical analyses were performed with MedCalc version 12.1.3.0 (MedCalc Software).

All tests were performed two-tailed and were considered as significant at *P* < 0.05. Comparison of analyzed parameters between two groups (cancers and control group) was performed by Mann-Whitney test because data did not follow normal distribution. Kruskal-Wallis test was used to compare analyzed parameters between three groups (cancers, benign lesions, and healthy tissues). The strength of the relationship between analyzed parameters was measured with Spearman’s correlation coefficient test.

## Results

**Fig. 1:**
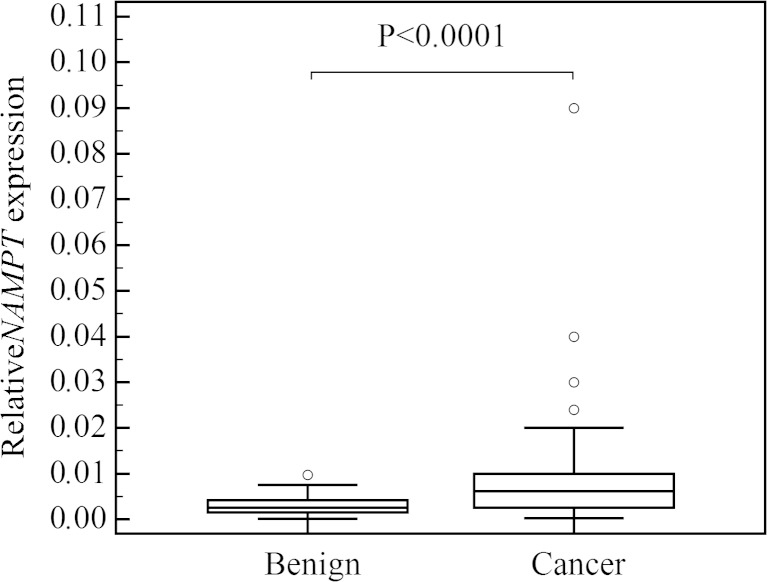
Comparison of relative NAMPT gene expression in benign and malignant thyroid nodules. *Central box* represents the values from the lower to upper quartile (25th to 75th percentile). The *middle line* represents the median. The thin vertical lines extending up or down from the boxes to horizontal lines (so-called whiskers) extend to a multiple of 1.5× the distance of the upper and lower quartile, respectively. Outliers are any values beyond the whiskers

**Fig. 2:**
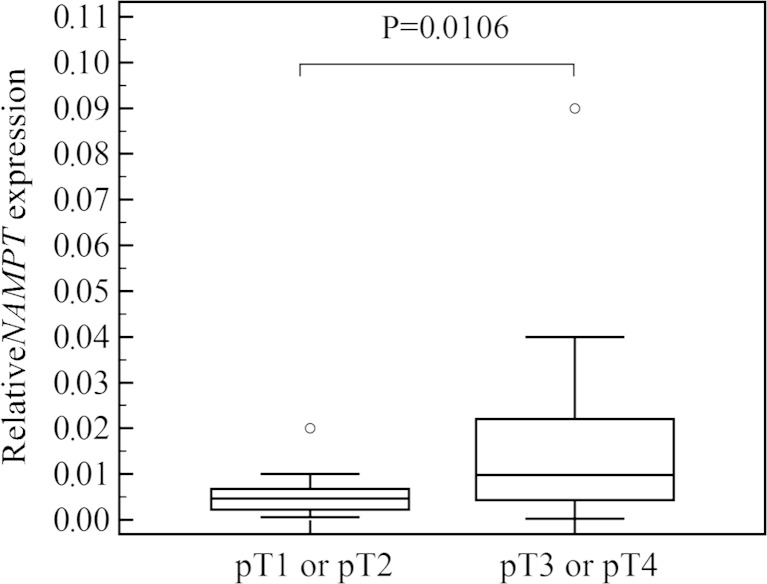
Comparison of relative *NAMPT* expression in tumors staged pT1 or pT2 and pT3 or pT4. *Central box* represents the values from the lower to upper quartile (25th to 75th percentile). The *middle line* represents the median. The thin vertical lines extending up or down from the boxes to horizontal lines (so-called whiskers) extend to a multiple of 1.5× the distance of the upper and lower quartile, respectively. Outliers are any values beyond the whiskers

The mean age of patients with thyroid cancer was 52 ± 17.5 years (range 18–81 years; 16 males and 24 females) and in the control group 51 ± 14 years (range 27–78 years; 18 males and 49 females). The study and the control groups did not differ according to patients’ sex and age. *NAMPT* expression was found in all analyzed tissue samples, and it was significantly higher in thyroid cancers than in the control group (*P* < 0.0001; Fig. 1). In multiple comparison analysis, *NAMPT* expression was higher in cancers than in benign lesions and healthy tissues (*P* = 0.0003), and there was no difference between benign lesions and healthy tissues. When papillary thyroid cancers (*n* = 29) were analyzed separately, *NAMPT* expression was also found to be higher than in the control group (*P* = 0.0002). *NAMPT* expression in thyroid cancers was positively correlated with tumor stage (*P* = 0.0012; *r* = 0.493), and it was significantly higher in tumors staged pT3 or pT4 (16 cases) than in tumors staged pT1 or pT2 (24 cases) (*P* = 0.0106; Fig. 2). Metastases to the lymph nodes were found in 12 out of 40 cases, and *NAMPT* expression was higher in the metastatic group (*P* = 0.0258). Multifocality was not associated with higher *NAMPT* expression (*P* = 0.3451). There was no association between patients’ age and *NAMPT* expression (*P* = 0.2234).

**Fig. 3:**
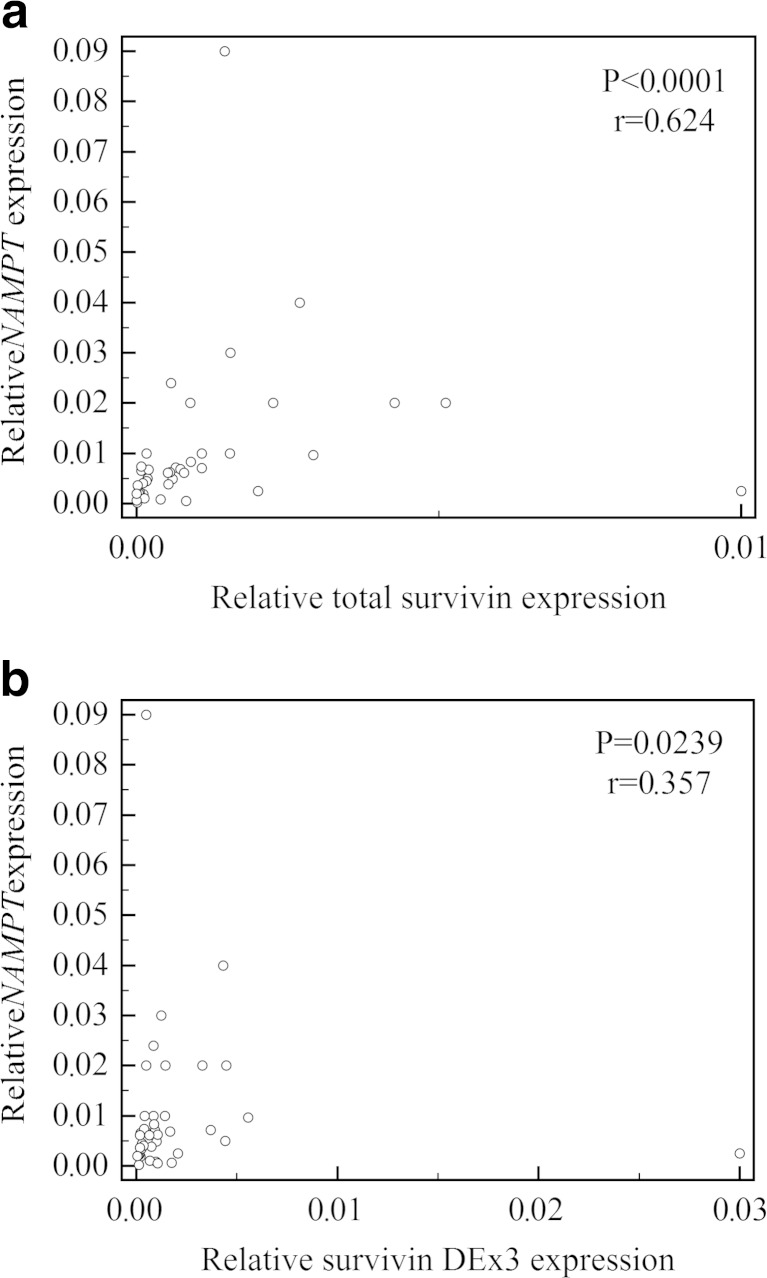
Positive correlations between relative *NAMPT* expression and total survivin (**a**), survivin DEx3 (**b**) expressions

*NAMPT* expression in thyroid cancers significantly correlated with survivin and with survivin splice variant DEx3 expressions (*P* < 0.0001; *r* = 0.624 and *P* = 0.0239; *r* = 0.357, respectively) (Fig. 3a, b). There was no correlation between *NAMPT* and survivin 2B expressions (*P* = 0.3508). The correlation between *NAMPT* and survivin/survivin DEx3/survivin 2B was not found in the control group (*P* = 0.3865/*P* = 0.9594/*P* = 0.5447, respectively).

## Discussion

To the best of our knowledge, this is the first study evaluating *NAMPT* expression in thyroid malignancy using quantitative RT-PCR. We found that *NAMPT* expression is significantly higher in thyroid cancers and it is correlated with tumor stage. Moreover, we observed higher *NAMPT* expression in cancers with lymph node involvement, suggesting that *NAMPT* might be a molecular marker of poor prognosis. Recent clinical studies have shown that elevated NAMPT expression is associated with poor survival in endometrial and breast cancers [26, 27].

NAMPT immunohistochemical expression was found to be increased in endometrial cancer and further increased in a subgroup with advanced tumor stage. However, the authors did not find increased NAMPT expression in patients with lymph node involvement [26]. Lee et al. revealed that visfatin expression in breast cancer positively correlated with tumor size [27]. Furthermore, elevated serum visfatin concentration has been evaluated as a negative prognostic factor in breast, endometrial, and colorectal cancers [26, 28–30].

The strong correlation between *NAMPT* and survivin expression in thyroid malignancies shown in our study also supports the hypothesis that high *NAMPT* expression might be a prognosis predictor for thyroid cancer. Moreover, we observed that *NAMPT* positively correlated with survivin splicing variant DEx3. Worthy of notice, survivin DEx3 overexpression is characteristic for aggressive thyroid cancers [23]. The correlation was not observed for survivin 2B, which is considered as a pro-apoptotic protein attenuating anti-apoptotic function of wild-type survivin [24]. Lack of correlation between *NAMPT* and survivin expressions neither in benign lesions nor in healthy tissues observed in our study further confirms different regulatory patterns of expression of both *NAMPT* and survivin in thyroid cancers. Survivin has been shown to inhibit apoptosis in G2/M phase. Survivin overexpression in cancer cells inhibits activity of caspase-3 and promotes cell survival [22]. Anti-apoptotic activity of NAMPT is related to upregulation of G1-S progression [31]. In neutrophils, NAMPT suppresses caspases 3, 8, and 9 and leads to the inhibition of apoptosis [32]. Based on the influence of survivin and NAMPT on the cell proliferation, the strong association between these two compounds might be explained. Understanding the relationship of NAMPT and survivin with the cancer cell cycle provides the potential therapeutic targets for novel inhibitors. NAMPT inhibitor CHS828/GMX1777 has shown antitumor activity in neuroendocrine tumors, including medullary thyroid carcinoma in nude mice [33]. CHS828/GMX1777 was an effective agent and a sensitizer for radiotherapy of head and neck squamous cell cancer model [34].

Recently, higher NAMPT immunohistochemical expression was found in the nuclear and cytoplasmic compartments in papillary and follicular thyroid cancers as compared with benign lesions [35]. Our results are consistent with these findings; however, our study group also included undifferentiated and medullary thyroid cancers.

Due to limited sample size, our study was underpowered to investigate the differences in *NAMPT* expression between certain histopathological types of thyroid cancer and this should be addressed in further studies. The strength of our study is that we analyzed the results not only from the molecular point of view, but we also correlated our findings with clinicopathological data.

## Conclusions

*NAMPT* expression is upregulated in thyroid malignancies. Elevated expression of *NAMPT* is associated with more advanced tumor stage and metastatic disease. *NAMPT* expression is correlated with survivin and survivin splicing variant DEx3 expressions. Further studies are needed to explain the role of NAMPT in thyroid cancer biology and to investigate the possible use of NAMPT inhibitors in thyroid malignancies.
